# Estimated pulse wave velocity is associated with all-cause and cardio-cerebrovascular disease mortality in stroke population: Results from NHANES (2003–2014)

**DOI:** 10.3389/fcvm.2023.1140160

**Published:** 2023-04-19

**Authors:** Huoming Huang, Xiancong Bu, Huachun Pan, Shumin Yang, Wenke Cheng, Quazi T. H. Shubhra, Niya Ma

**Affiliations:** ^1^ Department of Cardiovascular Medicine, Nanchang People's Hospital (The Third Hospital of Nanchang), Nanchang, China; ^2^ Department of Neurology, Zaozhuang Municipal Hospital, Zaozhuang, China; ^3^ College of Veterinary Medicine, Huazhong Agricultural University, Wuhan, China; ^4^ State Key Laboratory of Agriculture Microbiology, College of Veterinary Medicine, Huazhong Agricultural University, Wuhan, China; ^5^ Medical Faculty, University of Leipzig, Leipzig, Germany; ^6^ Stomatology Hospital of Guangzhou Medical University, Guangzhou Medical University, Guangzhou, China; ^7^ Translational Medicine Engineering Research Center of Guangdong Province, Foshan First People's Hospital, Foshan, China

**Keywords:** arterial stiffness, estimated pulse wave velocity, cardio-cerebrovascular disease mortality, all-cause mortality, stroke

## Abstract

**Background:**

Arterial stiffness is a significant determinant and evaluation of cardio-cerebrovascular disease and all-cause mortality risk in the stroke population. Estimated pulse wave velocity (ePWV) is a well-established indirect measure of arterial stiffness. We examined the association of ePWV with all-cause and cardio-cerebrovascular disease (CCD) mortality in the stroke population in a large sample of US adults.

**Methods:**

The study design was a prospective cohort study with data from the National Health and Nutrition Examination Survey (NHANES) from 2003 to 2014, between the ages of 18–85 years, with follow-up through December 31, 2019. 1,316 individuals with stroke among 58,759 participants were identified and ultimately, 879 stroke patients were included in the analysis. ePWV was calculated from a regression equation using age and mean blood pressure according to the following formula: ePWV = 9.587 − (0.402 × age) + [4.560 × 0.001 × (age^2^)] − [2.621 × 0.00001 × (age^2^) × MBP] + (3.176 × 0.001 × age × MBP) − (1.832 × 0.01 × MBP). Survey-weighted Cox regression models were used to assess the association between ePWV and all-cause and CCD mortality risk.

**Results:**

The high ePWV level group had a higher increased risk of all-cause mortality and CCD mortality compared to the low ePWV level group after fully adjusting for covariates. With an increase in ePWV of 1 m/s, the risk of all-cause and CCD mortality increased by 44%–57% and 47%–72% respectively. ePWV levels were linearly correlated with the risk of all-cause mortality (*P* for nonlinear = 0.187). With each 1 m/s increase in ePWV, the risk of all-cause mortality increased by 44% (HR 1.44, 95% CI: 1.22–1.69; *P *<* *0.001). When ePWV was <12.1 m/s, an increase in ePWV per 1 m/s was associated with a 119% (HR 2.19, 95% CI: 1.43–3.36; *P *<* *0.001) increase in CCD mortality risk; when ePWV was ≥12.1 m/s, an increase in ePWV per 1 m/s was not associated with in CCD mortality risk.

**Conclusion:**

ePWV is an independent risk factor for all-cause and CCD mortality in stroke patients. Higher levels of ePWV are associated with higher all-cause mortality and CCD mortality in stroke patients.

## Introduction

Arterial stiffness, also known as arterial elasticity loss, has been identified as a reliable indicator of altered arterial structure and function (1, 2). It is a significant predictor of cardiovascular events and all-cause mortality in asymptomatic people who have no overt cardiovascular disease (3–8). In patients with acute cerebrovascular disease, the infarct subtype was associated with increased arterial stiffness (9, 10). Currently, stroke remained the world's second-leading cause of death and the third-leading cause of death and disability in 2019 (11). The burden of stroke is manifested not only in the high mortality but also in high morbidity, which results in up to 50% of survivors being permanently disabled (12). Thus, stroke prevention *via* early intervention is essential. Aortic or carotid stiffness may improve stroke prediction and prognostic outcomes beyond another conventional risk factors (7, 13–15). It is extremely crucial to identify unknown stroke risk factors, particularly biomarkers of artery injury, to intervene appropriately. As a result, it is essential to develop a simple and easy-to-use tool for assessing arterial stiffness in clinical practice.

The gold standard for arterial stiffness has been established as the carotid-femoral pulse wave velocity (cfPWV) (13). Although the cfPWV measurement has been standardized (3), it requires costly and specialized equipment that is rarely available in clinical practice (14). Additionally, preceding research showed that age and blood pressure (BP) are the primary determinants of arterial stiffening in adults (16, 17). And Determinants of cfPWV progression and accelerated arterial aging in hypertensive patients were largely explained by age and BP values (18). Thus, as a strategy to overcome the limitations of evaluating aortic stiffness using cfPWV, researchers developed the concept of estimated pulse wave velocity (ePWV), which is calculated using an algorithm that takes into account age and means blood pressure (MBP) (15). Preliminary research found a strong correlation between ePWV and measured cfPWV (15), implying that daily ePWV measurements can be used to monitor the severity of arterial stiffness. Furthermore, previous research has discovered that ePWV has an incremental predictive value in Western populations for stroke, myocardial infarction, cardiovascular mortality, and other outcomes (19–21). Therefore, we investigated the long-term association between ePWV in assessing arterial stiffness and all-cause and CCD mortality in the stroke population.

## Method

### Study design and population

The study design was a prospective cohort study with data from the National Health and Nutrition Examination Survey (NHANES) from 2003 to 2014, followed through the end of December 31, 2019. The survey, which includes interviews, physical examinations at home or mobile examination centers, and laboratory tests, is administered by the National Centre for Health Statistics and follows a complex, stratified, multi-stage probabilistic design. The survey is conducted on a two-year cycle. Detailed sampling methods and data collection processes have been published elsewhere (22). NHANES was administered by the National Center for Health Statistics of the US Centers for Disease Control and Prevention (CDC) and approved by the NHANES Institutional Review Board. All participants provided the necessary written informed consent.

A total of 58,759 participants participated in six cycles of the NHANES between 2003 and 2014. 1,316 stroke population were identified using standardized questionnaires (Has a doctor or other health professional ever told you that you had a stroke?/How old were you when you were first told had a stroke?). One of the participants was aged less than 17 years, one was pregnant, two had missing follow-up data, 274 had cancer, and 75 had no ePWV data were excluded. Meanwhile, to reduce the potential reverse causation bias, participants who died within 2 years of follow-up were excluded, and ultimately, 879 participants were included in the analysis. Detailed information is available at https://wwwn.cdc.gov/Nchs/Nhanes/.

### Evaluation of ePWV

ePWV is calculated according to the following formula described by Greve et al. (15). The equation described was derived from the Reference Values for Arterial Stiffness Collaboration (16).

$$\mathrm{ePWV}\;=\;.587-(0.402\times \mathrm{age})+[4.560\times 0.001\times (\mathrm{ag}e^{2})]\text{–}[2.621\times 0.00001\times (\mathrm{ag}e^{2})\times \mathrm{MBP}]+(3.176\times 0.001\times \mathrm{age}\times \mathrm{MBP})\text{–}(1.832\times 0.01\times \mathrm{MBP})$$

In this formula, age is measured in years, and mean blood pressure (MBP) is calculated as diastolic blood pressure (DBP) +0.4× [systolic blood pressure (SBP)-DBP]. Blood pressure is measured using a uniform sphygmomanometer. Before the test, participants were placed in a quiet sitting position for five minutes. Trained inspectors carry out the above operations. The blood pressure value is the average of at least three measurements.

### Study endpoints

The outcomes of this study were all-cause and CCD mortality. All-cause mortality was the total of all mortality, and CCD mortality was diagnosed by the International Classification of Diseases version 10 code [ICD-10 I00–I09, I11, I13 or I20–I51, Cerebrovascular diseases (I60–I69)].

### Assessment of other variables

Information on age, sex, race, education level, marriage, family income, smoking and drinking status, medical history, and medication use was collected from family interviews and mobile examination centers using standardized questionnaires. Biochemical indicators are tested through a rigorous procedure, which can be found in the NHANES Laboratory/Medical Technician Procedure Manual (22).

In addition, to facilitate data integration, we further classified the following variables: Age (≤60, >60 years), race (non-Hispanic white, non-Hispanic blacks and Mexican Americans, or others), an education level (less than grade 9, 9–11 grade/graduated from high school or equivalent, college graduated or above), marital status (never married, married/separated, divorced/widowed/living with partner/others). Besides, smoking status was classified as never smoking (smoking <100 cigarettes/session), former smoking (smoking >100 cigarettes/session, now not smoking at all), and current smoking (smoking >100 cigarettes/session, now smoking some days or every day) (23). Drinking alcohol into never drinking (life <12 drinks), was drinking (alcohol or 12 drinks in the life, but not drinking) last year, the mild/moderate drinkers (over the past year women are drinking 1 time/day or less, men drink 2 times/day or less), heavy drinkers (over the past year women drinking >1 time/day, men drinking >2 times/day) (24).

### Statistical analysis

Appropriate weighting (Mec2yr weights) was carried out in the statistical analysis. In population baseline characteristics, continuous variables are expressed as weighted means (standard errors, SE) and categorical variables as unweighted counts (weighted %). Spearman's correlation coefficient was used for the assessment of the relationship between ePWV and age. Hazard ratios (HRs) and 95% confidence intervals (CIs) of ePWV with all-cause and CCD mortality were assessed using survey-weighted Cox regression models. In the Cox model, ePWV was analyzed as a categorical (low vs. group) and a continuous variable (1 m/s), respectively. From baseline characteristics, confounders were selected based on their association with the outcome of interest or a change in the effect estimate of more than 10% (25). Supplementary Table S1 shows the variables contributing more than 10% to each result. Meanwhile, a time-dependent Receiver operating characteristic (ROC) curve was used to assess the predictive value of ePWV for all-cause and CVD mortality. Furthermore, we obtained five data sets by multiple imputations for the missing data, and the pooled multivariate Cox regression results were regarded as a sensitivity analysis.

Subgroup analysis was performed according to the following clinical characteristics: sex (male, female), age (<60, ≥60 years), BMI (<30, ≥30 kg/m^2^), race (non-Hispanic white, non-Hispanic black, Mexican American, and others), heart attack (no/yes), and history of hypertension (no/yes), and *P* values for interaction were obtained. In addition, a generalized additive model (GAM) was used to visually assess the dose-dependent relationship between ePWV and the risk of mortality (26), and log-likelihood ratio tests the *P*-values for nonlinear. If the nonlinear association is observed, a two-piecewise linear regression model is performed to calculate the inflection point where the ratio of ePWV to mortality significantly changes in the smooth curve (1).

All statistical analyses were performed by R software (http://www.R-project.org, The R Foundation), GraphPad Prism (Version 9.0; USA, San Diego, CA), and EmpowerStats (Version 4.2.0, www.R-project.org, X&Y Solutions, Inc., Boston, MA). *P*-values less than 0.05 indicate statistically significant differences.

## Results

**Figure 1:**
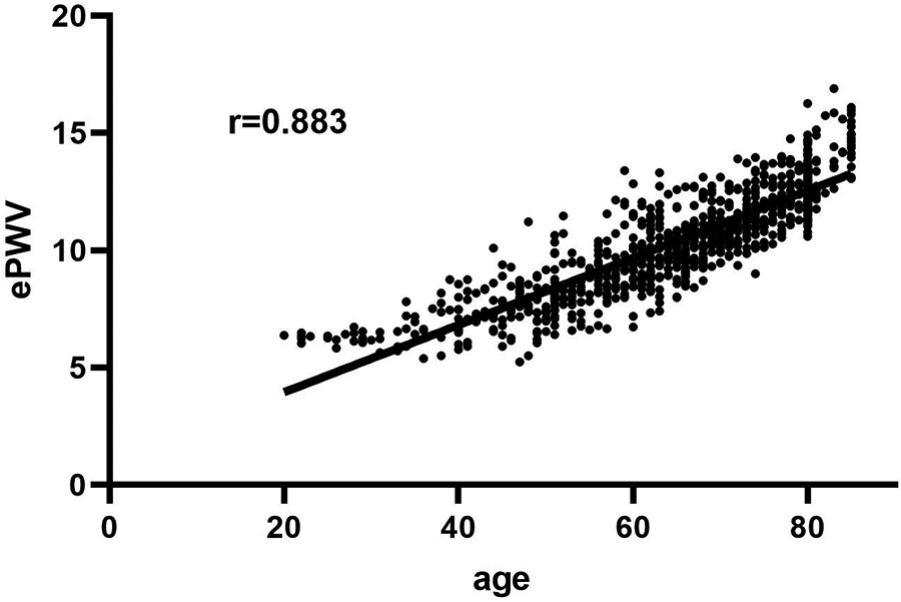
Correlations between age and ePWV.

A total of 58,759 individuals participated in NHANES from 2003 to 2014, and 1,316 cases of stroke were identified. Among these stroke patients, one participant was under 20 years of age, one was pregnant, two had missing follow-up data, 274 had cancer, and 75 did not have ePWV data. Besides, 84 died within the first two years of follow-up were further excluded, and 879 stroke patients were eligible for analysis, representing a stroke population of 4,543,943. The 879 stroke patients were followed up for 7,706 person-years and divided into low (5.24–10.37 m/s) and high groups (10.38–16.89 m/s) based on median ePWV levels. The detailed baseline characteristics of the 879 stroke patients were summarized in Table 1. ePWV levels were significantly and positively correlated with age (*r* = 0.883), as shown in Figure 1.

**Table 1:** Survey-weighted baseline characteristics of stroke patients (representing 4,543,943 individuals) stratified by ePWV median levels.

Variables	Low ePWV (5.24–10.37 m/s)	High ePWV (10.38–16.89 m/s)	Variables	Low ePWV (5.24–10.37 m/s)	HighePWV (10.38–16.89 m/s)
	*n* = 439	*n* = 440		*n* = 439	*n* = 440
**Represented size**	2,678,800	1,865,144	**Osteoporosis**		
**BMI (kg/m^2^)**	30.36 (0.43)	29.07 (0.39)	No	314 (91.16)	303 (81.75)
**PIR**	2.35 (0.11)	2.33 (0.09)	Yes	26 (8.84)	58 (18.25)
**Waist (cm)**	104.52 (1.07)	102.0 (0.94)	**Arthritis**		
**HB (g/dl)**	14.13 (0.11)	13.68 (0.11)	No	224 (52.79)	191 (41.58)
**HBA_1c_ (%)**	6.07 (0.09)	6.04 (0.06)	Yes	215 (47.21)	247 (58.42)
**FPG (mg/dl)**	115.12 (3.52)	120.73 (3.87)	**DM**		
**AST (U/L)**	25.59 (0.61)	25.06 (0.51)	No	292 (71.21)	224 (58.28)
**ALT (U/L)**	24.61 (0.83)	21.13 (0.61)	Yes	147 (28.79)	196 (41.72)
**TB (µmol/L)**	11.37 (0.30)	12.42 (0.29)	**CHD**		
**Creatinine (µmol/L)**	87.72 (2.85)	97.94 (2.62)	No	383 (87.67)	348 (80.69)
**HDL (mmol/L)**	1.30 (0.03)	1.38 (0.03)	Yes	51 (12.33)	80 (19.31)
**TC (mmol/L)**	4.98 (0.07)	4.94 (0.07)	**Hyperlipidemia**		
**LDL (mmol/L)**	2.90 (0.09)	2.82 (0.10)	No	86 (19.02)	71 (14.42)
**TG (mmol/L)**	1.77 (0.13)	1.63 (0.08)	Yes	353 (80.98)	369 (85.58)
**Age**			**Hypertension**		
<60 years	259 (65.15)	13 (3.31)	No	118 (31.03)	41 (9.15)
≥60 years	180 (34.85)	427 (96.69)	Yes	321 (68.97)	399 (90.85)
**Gender**			**Antihypertensive medication**		
Women	232 (53.92)	227 (60.50)	No	391 (90.90)	367 (81.23)
Men	207 (46.08)	213 (39.50)	Yes	51 (9.10)	73 (18.77)
**Race**			**Diabetes medications**		
Non-hispanic white people	182 (64.91)	235 (73.72)	No	332 (79.73)	303 (71.72)
Non-hispanic black people	139 (17.68)	105 (13.35)	Yes	107 (20.27)	137 (28.28)
Mexican Americans	64 (6.70)	52 (4.31)	**Alcohol user**		
Other races	54 (10.71)	48 (8.63)	Never	43 (6.67)	98 (24.29)
**Education Levels**			Former	143 (32.12)	175 (35.58)
Less than 9th grade	57 (8.41)	106 (16.95)	Mild/moderate	97 (23.28)	96 (25.36)
9–11th grade/high school grade or equivalent	214 (46.18)	188 (44.28)	Heavy	122 (29.59)	31 (6.81)
College graduate or above	168 (45.41)	143 (38.76)	Refused/Don't know	34 (8.34)	40 (7.96)
**Marital status**			**Smoke status**		
Never married	55 (11.50)	18 (3.19)	Never	156 (36.03)	206 (49.85)
Married/Separated	233 (55.48)	223 (54.31)	Former	115 (25.36)	173 (36.91)
Divorced/Widowed/Living with partner/others	151 (33.02)	199 (42.05)	Current	168 (38.60)	61 (13.24)
**Asthma**			** **		
No	334 (75.03)	383 (86.86)			
Yes	104 (24.97)	56 (13.14)			

Continuous variables are expressed as weighted mean (standard error, SE). Categorical variables are expressed as counts (weighted %). ePWV, estimated pulse wave velocity; PIR, poverty income ratio; CHF, coronary heart failure; BMI, body mass index; HB, hemoglobin; FPG, fasting plasma glucose; TB, total bilirubin; TC, total cholesterol; LDL, low-density lipoprotein cholesterol; TG, triglycerides; HDL, high-density lipoprotein cholesterol; ALT, alanine aminotransferase; AST, aspartate aminotransferase; HbA_1c_, glycated hemoglobinA1c.

### ePWV and all-cause mortality

In the crude model, the higher-level group had a 2.8-fold (HR 3.8, 95% CI: 2.81–5.13; *P *< 0.001) increased risk of all-cause mortality compared to the lower ePWV level group (Table 2). With an increase in ePWV of 1 m/s, the risk of all-cause mortality increased by 44% (HR 1.44, 95% CI: 1.36–1.52; *P *<* *0.001). In Model 1, age, race, and gender were adjusted and the results remained stable. In Model 2, after full adjustment for maximum variables, the risk of all-cause mortality increased 3.75-fold (HR 4.75, 95% CI: 1.95–11.58; *P* = 0.001) in the higher-level group compared to the lower ePWV level group. Similarly, the risk of all-cause mortality increased by 57% (HR 1.57, 95% CI: 1.29–1.90; *P *< 0.001) with an increase in ePWV of 1 m/s (Table 2). Besides, the results of ePWV and the risk of all-cause mortality remained significantly positively correlated before and after multiple imputations (Supplementary Table S2).

**Table 2:** Weighted univariate and multivariate Cox regression to assess the association between ePWV levels and the risk of all-cause and cardiovascular disease mortality in stroke patients.

	Low ePWV (5.24–10.37 m/s)	High ePWV (10.38–16.89 m/s)	*P*-value	Every 1 m/s increase in ePWV	*P*-value
**All-cause mortality**					
Number of deaths	107	262		369	
Crude model	1	3.80 (2.81–5.13)	<0.001	1.44 (1.36–1.52)	<0.001
Model 1^a^	1	2.01 (1.38–2.93)	<0.001	1.33 (1.23–1.43)	<0.001
Model 2^b^	1	4.75 (1.95–11.58)	0.001	1.57 (1.29–1.90)	<0.001
**CCD mortality**					
Number of deaths	39	105		144	
Crude model	1	3.97 (2.60–6.07)	<0.001	1.47 (1.34–1.62)	<0.001
Model 1^a^	1	2.02 (1.22–3.33)	0.006	1.36 (1.22–1.52)	<0.001
Model 2^c^	1	5.01 (1.31–19.22)	0.019	1.72 (1.23–2.40)	0.001

^a^ Model 1 adjust age, race, and gender.

^b^ Model 2 adjust age, BMI, gender, race, education levels, marital status, waist, HB, HBA1c, FPG, ALT, AST, TB, creatinine, asthma, DM, CHD, Hypertension, diabetes medications, alcohol use, smoke.

^c^ Model 2 adjusts age, BMI, gender, race, education levels, marital status, HB, HBA1c, FPG, ALT, TB, creatinine, HDL, LDL, arthritis, DM, CHD, diabetes medications, and alcohol use.

**Figure 2:**
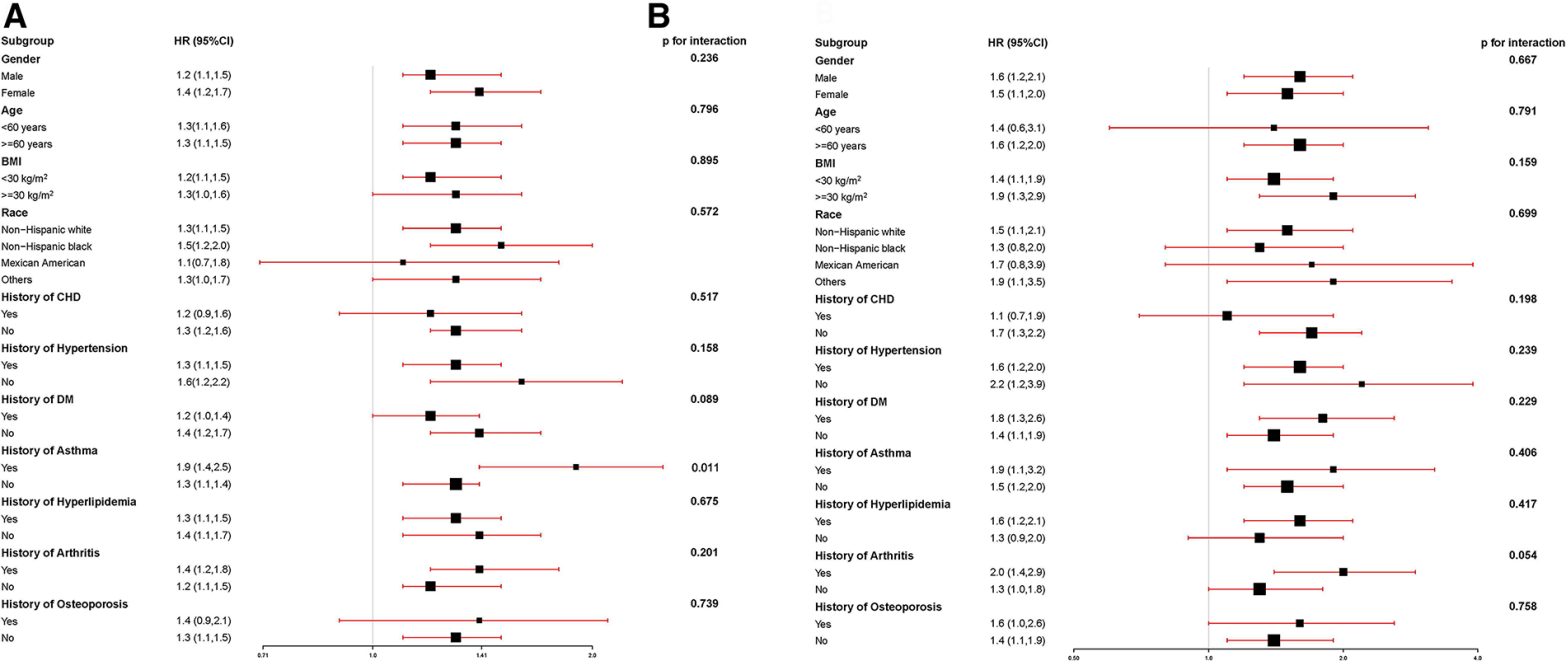
(**A**) The association between ePWV levels and all-cause risk in the stroke population; (**B**) the association between ePWV levels and CCD mortality risk in the stroke population.

To assess the robustness of the association between ePWV levels and all-cause risk in the stroke population, the subgroup analysis was performed, as shown in Figure 2A. With each 1 m/s increase in ePWV, the risk of all-cause mortality correspondingly increased by 20%–90%. For every 1 m/s increase in ePWV, those with a history of asthma had a higher risk of all-cause mortality (HR 1.9, 95% CI: 1.40–2.50; *P* = 0.011) than those without a history of asthma.

### ePWV and CCD mortality

In the crude model, the higher level group had a 2.97-fold (HR 3.97, 95% CI: 2.60–6.07; *P *<* *0.001) increased risk of CCD mortality compared to the lower ePWV level group (Table 2). With an increase in ePWV of 1 m/s, the risk of CCD mortality increased by 47% (HR 1.47, 95% CI:1.34–1.62; *P *< 0.001). In Model 1, age, race, and gender were adjusted and the results remained stable. In Model 2, after full adjustment for maximum variables, the risk of CCD mortality increased 4.01-fold (HR 5.01, 95% CI: 1.31–19.22; *P* = 0.019) in the higher-level group compared to the lower ePWV level group. Similarly, the risk of CCD mortality increased by 72% (HR 1.72, 95% CI: 1.23–2.40; *P* = 0.001) with an increase in ePWV of 1 m/s (Table 2). Besides, the results of ePWV and the risk of CCD mortality remained significantly positively correlated before and after multiple imputations (Supplementary Table S2).

To assess the robustness of the association between ePWV levels and CCD mortality risk in the stroke population, the subgroup analysis was performed, as shown in Figure 2B. With each 1 m/s increase in ePWV, the risk of CCD mortality correspondingly increased by 10%–100%.

### Dose-dependent relationship between ePWV levels and risk of all-cause and CCD mortality

**Figure 3:**
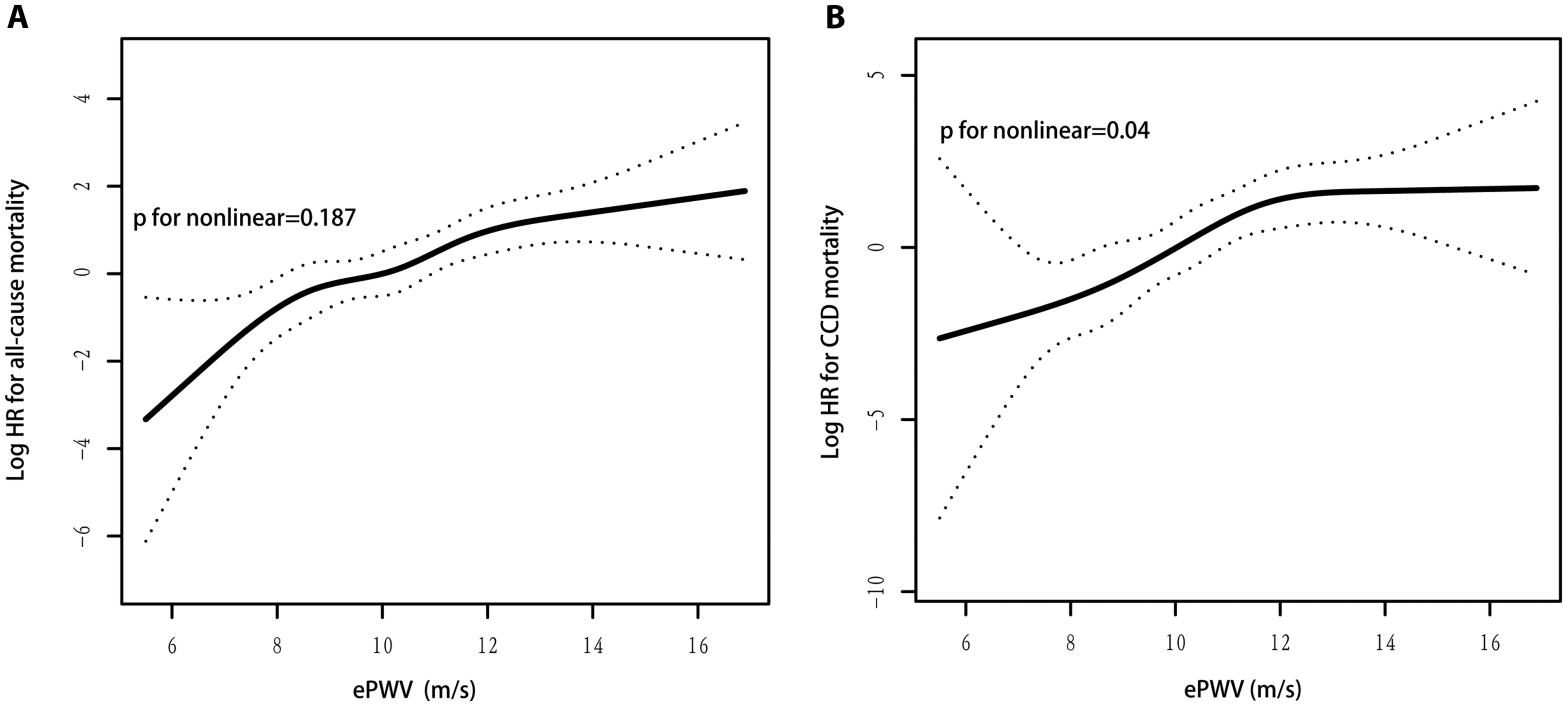
(**A**) ePWV levels were linearly correlated with the risk of all-cause mortality; (**B**) ePWV levels were nonlinearly correlated with the risk of CCD mortality.

As shown in Figure 3A, ePWV levels were linearly correlated with the risk of all-cause mortality (*P* for nonlinear = 0.187). With each 1 m/s increase in ePWV, the risk of all-cause mortality increased by 44% (HR 1.44, 95% CI: 1.22–1.69; *P *< 0.001), which is approximately the same as the results of the Cox regression model.

As shown in Figure 3B, ePWV levels were nonlinearly correlated with the risk of CCD mortality (*P* for nonlinear = 0.04). The two-piecewise linear regression model showed an inflection point of 12.1 m/s for ePWV and CCD mortality risk. When ePWV was 12.1 m/s, an increase in ePWV per 1 m/s was associated with a 119% (HR 2.19, 95% CI: 1.43–3.36; *P *< 0.001) increase in CCD mortality risk; when ePWV was ≥12.1 m/s, an increase in ePWV per 1 m/s was not associated with in CCD mortality risk (HR 0.99, 95% CI: 0.58–1.69; *P *= 0.966) (Table 3). In addition, because the *P* value for nonlinear is nearly 0.05, the linear results were also reported. With each 1 m/s increase in ePWV, the risk of CCD mortality increased by 55% (HR 1.55, 95% CI: 1.21–1.98; *P *<* *0.001), which is approximately the same as the results of the Cox regression model.

**Table 3:** The results of two-piecewise linear regression model for ePWV and the risk of all-cause and CCD mortality in stroke patients.

Outcome	Inflection-point of ePWV (m/s)	HR	95% CI	*P*-value
CCD mortality	<12.1	2.19	1.43–3.36	<0.001
	≥12.1	0.99	0.58–1.69	0.966

HRs has been fully adjusted as described in the Table 2.

### Predictive value of ePWV for 10-year all-cause and CCD mortality

**Figure 4:**
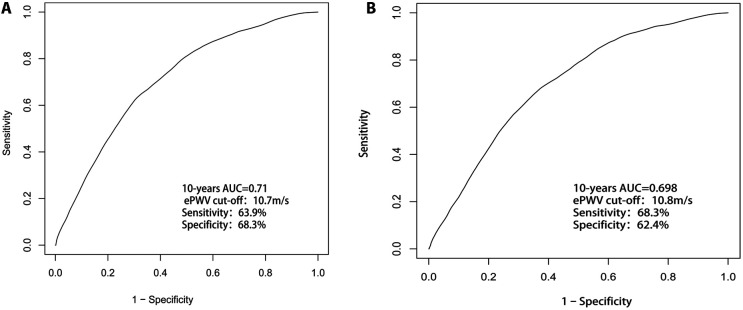
(**A**) ePWV had a strong predictive value (AUC = 0.71) for 10-year all-cause mortality in the stroke population; (**B**) ePWV maintained a strong predictive value (AUC = 0.698) for 10-year CCD mortality in the stroke population.

As shown in Figure 4A, ePWV had a strong predictive value (AUC = 0.71) for 10-year all-cause mortality in the stroke population, with a cutoff value of 10.7 m/s, and a sensitivity and specificity of 63.9% and 68.3%, respectively. Similarly, as shown in Figure 4B, ePWV maintained a strong predictive value (AUC = 0.698) for 10-year CCD mortality in the stroke population, with a cutoff value of 10.8 m/s, and a sensitivity and specificity of 68.3.% and 62.4%, respectively.

## Discussion

The results of our research can be summarized in two aspects. (1) Higher levels of ePWV were associated with all-cause and CCD mortality in stroke patients. (2) ePWV was linearly related to all-cause mortality and non-linearly related to CCD.

Several previous studies have revealed a link between ePWV level and the risk of all-cause and CCD mortality in normal and diseased populations. In hypertensive adults (27) and the general population, ePWV is linked to cardiovascular outcomes and all-cause mortality (19–21). Vlachopoulos and colleagues reported that the ePWV predicted the primary composite cardiovascular outcome with an HR of 1.30 (95% CI: 1.17–1.43; *P* < 0.001) (28) and all-cause mortality with an HR of 1.65 (95% CI: 1.46–1.86; *P* < 0.001) (27). Chunpeng Ji and colleagues also discovered that the ePWV was associated with the risk of CVDs and all-cause mortality regardless of cardiovascular risk factors (29). Greve and colleagues (15) found that the ePWV and measured cfPWV predicted the combined outcome of nonfatal myocardial infarction, cardiovascular mortality, ischemic heart disease, and stroke. In the other study, they also found that ePWV is independently associated with the risk of stroke in middle-aged men (21). Thus, ePWV may be a simple metric generated from age and BP that may offer insight into all-cause and CCD mortality risk in the stroke population and be used in clinical practice.

According to our studies, the stroke population with arterial stiffness and a high ePWV level had an increased risk of all-cause and CCD mortality with increasing ePWV. Whether variables were adjusted or not, the mortality rate of all-cause and CCD was still higher in the stroke population with high ePWV values than in those with low ePWV values. Furthermore, each 1 m/s increase in ePWV increased the risk of all-cause and CCD mortality in the stroke population. Apart from an increase in ePWV per 1 m/s, when ePWV was 12.1 m/s, there was no association with CCD mortality risk. We considered that the stroke population may be complicated by hypertension as arterial stiffness increases and that most of the population is already on antihypertensive therapy at this time. Because ePWV is calculated using age and blood pressure, it may be influenced by antihypertensive therapy in predicting the risk of death from CCD. Besides, our study provided some evidence that ePWV is associated with all-cause and CCD mortality in the stroke population. As demonstrated by some previous studies, revealed that high PWV doubles the risk of future ischemic stroke in a population with acute lacunar infarction, regardless of age, gender, or blood pressure levels (28). Measuring PWV during the acute phase of an ischemic stroke is useful for predicting future ischemic stroke (28). As a result, Aortic pulse wave velocity (PWV) is a well-established indirect indicator of arterial stiffness (30). Although ePWV is not a replacement for cfPWV, it can be used in conjunction with traditional risk classification to improve risk prediction in the stroke population when measuring cfPWV is not feasible. Furthermore, the use of ePWV will lead to a greater understanding of the role of arterial stiffness and will assist physicians in incorporating it into clinical practice. Second, in some populations with arterial stiffness, ePWV may be used to assess the efficacy of antihypertension treatment.

The association of arterial stiffness with mortality in stroke patients may involve several mechanisms. Arterial stiffness is substantially influenced by blood pressure, and angiogenic hypertension indices have been associated with cancer (31). It also is linked to inflammation and oxidative stress (32, 33), which play a role in the pathophysiology of high-mortality diseases such as cancer and inflammatory diseases. Vascular biomarkers are strongly linked to genetic indicators of biological aging and life expectancy, indicating a genetic predisposition to arterial function and death (34, 35). Increased arterial stiffness also causes hypertension and high pulse pressure, reduces coronary perfusion pressure, and increases left ventricular afterload, promoting remodeling and dysfunction (36). Increased pulse pressure enhances pulsatile flow penetration into organ microvasculature, including the brain, heart, and kidney (37). Hemodynamic stress, pulsatile pressure, and blood pressure variability damage the brain and heart (38).

### Perspectives

To date, studies on the correlation between ePWV and cfPWV are limited. American adults with mild-to-moderately increased BP and obesity had a high association between baseline ePWV and cfPWV (*r* = 0.70) (39). In Danish, French, Australian, and US adults, there is a weak to moderate association between ePWV and cfPWV (*r* varied from 0.35 to 0.66) (15, 40, 41).

Currently, CfPWV remains the gold standard for the assessment of arterial stiffness. Assessment of the cfPWV still requires relatively sophisticated technical skills and equipment. ePWV is more affordable and simpler to operate than cfPWV. Because of these advantages, ePWV may be applied more, particularly in community hospitals without equipment, in rural areas, and for a bigger population, especially in developing countries. ePWV could be a screening tool and “gatekeeper” for magnetic resonance imaging of aortic stiffness (42). ePWV predicts cardiovascular and cerebrovascular events and all-cause mortality independent of traditional CVD risk factors (15, 19–21, 40, 42–47). Furthermore, they revealed that ePWV is linked to recognized indicators of vascular aging and could serve as a valuable tool for advancing research on vascular aging in their recent study by Heffernan KS et al. (48). Therefore, ePWV could be a candidate for the initial assessment of arterial stiffness and the early identification of those at high risk. This will help this population to be evaluated and treated for arterial stiffness as early as possible to reduce the risk of mortality.

### Strengths and limitations

The Strengths of our study include the large, community-based cohort with high retention, the standardized and over whole data collection database, and the inclusion of potential confounders such as BMI, Race, Marital Status, Education Levels Gender, household income, health behaviors, and serum concentrations of glucose and blood lipids into the multivariable analysis.

Our study had the following limitations: (1) Due to the design of this observational study, a causal relationship between ePWV and mortality risk cannot be inferred. (2) The influence of other covariates. (3) The research population is a stroke population from the United States, and may not apply to other populations. (4) Due to a lack of relevant data, we did not investigate BP variability and changes in BP-lowering treatment. (5) We did not categorize the stroke population in detail and evaluated the associations between ePWV and all-cause and CCD mortality risk in different stroke subtypes.

## Conclusion

ePWV is an independent risk factor for all-cause and CCD mortality in stroke patients. Higher levels of ePWV are associated with higher all-cause mortality and CCD mortality in stroke patients.

## Data Availability

Publicly available datasets were analyzed in this study. This data can be found here: https://www.cdc.gov/nchs/nhanes/index.htm.
