# Supplementary material for: Estimated pulse wave velocity is associated with all-cause and cardio-cerebrovascular disease mortality in stroke population: Results from NHANES (2003–2014)
Source: Front Cardiovasc Med. 2023 Apr 19;10:1140160. doi: 10.3389/fcvm.2023.1140160 (PMC10154635; doi:10.3389/fcvm.2023.1140160)
Supplement: Supplementary file 1 [file Table1.docx]

**Table S1. Selected covariates**

| **Y** | **X** | **Selected based on criteria 1** | **Selected based on criteria 2** |
| --- | --- | --- | --- |
| All-cause  Mortality | ePWV  Dichotomous | Age, Education levels, Waist, ALT, AST | Age, BMI, Gender, Race, Education levels, Marital Status, Waist, HB, HBA1c, FPG, ALT, AST, TB, Creatinine, Asthma, DM, CHD, Hypertension, Diabetes medications, Alcohol use, Smoke |
| CCD Mortality | ePWV Dichotomous | Age, BMI, Education levels, HB, HBA1c, FPG, HDL, Arthritis, CHD | Age, BMI, Gender, Race, Education levels, Marital Status, HB, HBA1c, FPG, ALT, TB, Creatinine, HDL, Arthritis, DM, CHD, Diabetes medications, Alcohol use |

**Notes:**

**Criteria 1：add the covariate to the basic model or remove it from the full model, change X coefficient. >10%.
Criteria 2：effect of criterion 1 or covariates on the p-value of the regression coefficient of Y < 0.1**

| **Table S2. Survey-weighted multivariate Cox regression** **performed to assess the ePWV levels and the risk of all-cause and CCD mortality after multiple imputation of 5 data sets.** | | | | | | |
| --- | --- | --- | --- | --- | --- | --- |
| **Low vs. High** | Multiple Imputation1 | Multiple Imputation 2 | Multiple Imputation 3 | Multiple Imputation 4 | Multiple Imputation 5 | **Pooled results** |
| **All-cause mortality** | 1.99 (1.38 to 2.86) | 1.93 (1.34 to 2.78) | 1.86 (1.28 to 2.70) | 1.96 (1.37 to 2.79) | 2.0 (1.39 to 2.89) | **1.94 (1.35 to 2.79)** |
| **CCD mortality** | 2.01 (1.15 to 3.51) | 2.01 (1.16 to 3.50) | 2.12 (1.23 to 3.65) | 2.28 (1.34 to 3.91) | 2.14 (1.23 to 3.73) | **2.09 (1.21 to 3.63)** |
| **Every 1m/s ePWV increase** |  |  |  |  |  |  |
| **All-cause mortality** | 1.33 (1.21 to 1.47) | 1.33 (1.21 to 1.46) | 1.34 (1.22 to 1.47) | 1.34 (1.22 to 1.47) | 1.34 (1.22 to 1.48) | **1.34 (1.22 to 1.47)** |
| **CCD mortality** | 1.37 (1.20 to 1.56) | 1.37 (1.20 to 1.56) | 1.38 (1.22 to 1.57) | 1.38 (1.22 to 1.57) | 1.38 (1.21 to 1.57) | **1.38 (1.21 to 1.57)** |

**HRs have been fully adjusted as described in Table 2.**
